# Supplementary material for: Predation and fragmentation portrayed in the statistical structure of prey time series
Source: BMC Ecol. 2009 May 6;9:10. doi: 10.1186/1472-6785-9-10 (PMC2689204; doi:10.1186/1472-6785-9-10)
Supplement: Additional file 2 — Voles and related classes ODDox Documentation. ODDox documentation of the agent-based model (ALMaSS) applied by Hendrichsen et al. The documentation is started by activating main.html. [file 1472-6785-9-10-S2.zip › Vole_ODDox/_genetic_material_8cpp.html]

ALMaSS ODDox: GeneticMaterial.cpp File Reference

- Main Page
- Related Pages
- Classes
- Files

# GeneticMaterial.cpp File Reference

---

## Detailed Description

**GeneticMaterial.cpp This file contains the source for the genetic material classes**   

by Chris J. Topping   
Version of 1st Nov 2000   
All rights reserved.   
  
With additions as noted in:   
Doxygen formatted comments in May 2008   

`#include "ls.h"`  
`#include "stdio.h"`  
`#include "GeneticMaterial.h"`  

|  |  |
| --- | --- |
|  |  |
| Defines | |
| #define | \_\_GENDEBUG |
| #define | \_CRT\_SECURE\_NO\_DEPRECATE |
| Variables | |
| const int | MutationChance = 1000 |

---

## Define Documentation

#define \_\_GENDEBUG

#define \_CRT\_SECURE\_NO\_DEPRECATE

---

## Variable Documentation

const int MutationChance = 1000

Referenced by GeneticMaterial::Mutation\_1(), GeneticMaterial::Mutation\_1ab(), GeneticMaterial::Mutation\_2(), and GeneticMaterial::Mutation\_3().

---

Generated on Thu Jan 22 14:13:45 2009 for ALMaSS ODDox by 
 1.5.6
